# Electric Bicycle Series Arc Fault Identification Method Based on Improved PCA and SVM

**DOI:** 10.3390/s26134018

**Published:** 2026-06-24

**Authors:** Kai Yang, Jiaqi Chen, Zuxuan Yang, Ziyu Ma, Rencheng Zhang

**Affiliations:** 1 Key Laboratory of Process Monitoring and System Optimization for Mechanical and Electrical Equipment (Fujian Provincial Department of Education), College of Mechanical Engineering and Automation, Huaqiao University, Xiamen 361021, China; cc7@stu.hqu.edu.cn (J.C.); yzx6666@stu.hqu.edu.cn (Z.Y.); maziyu@stu.hqu.edu.cn (Z.M.); phzzrc@hqu.edu.cn (R.Z.); 2 Fujian Key Laboratory of Green Intelligent Drive and Transmission for Mobile Machinery, Huaqiao University, Xiamen 361021, China

**Keywords:** electric bicycle, series arc fault, principal component analysis, support vector machine, time–frequency characteristics

## Abstract

Electric bicycles are popular due to their environmental benefits and convenience. However, electric bicycle fires caused by series arc faults remain a serious safety concern. This study focuses on series arc fault identification for electric bicycles under complex operating conditions, covering state of charge (SoC), torque, and speed variations, and simultaneously considers normal state, DC-side series arc fault, and AC-side series arc fault conditions. Five time-domain features, namely root mean square (RMS), standard deviation (STD), skewness (SK), kurtosis (KUR), and current amplitude (CA), and three frequency-domain features, namely amplitude–frequency energy (AFE), amplitude–frequency mean (AFM), and amplitude–frequency kurtosis (AFK), are extracted. An improved principal component analysis (PCA)-based feature fusion method transforms the eight original time–frequency features into a five-dimensional PCA-fused feature representation consisting of PC1, PC2, PC3, fused PC4–PC7, and PC8. The fused features are classified using a radial basis function (RBF)-support vector machine (SVM) model. The proposed method achieves 98.68% test accuracy, 0.9869 Macro-F1, and 0.9931 Macro-AUC. A classifier comparison and feature-level latency analysis are also provided to clarify the accuracy–cost tradeoff and deployment feasibility. The results indicate that the proposed method can provide an interpretable and lightweight solution for electric bicycle controllers, battery management systems (BMSs), and onboard safety-monitoring applications.

## 1. Introduction

Electric bicycles are rapidly gaining popularity worldwide due to their environmental benefits and convenience. According to Statista Research [1], global electric bicycle sales reached approximately 42 million units in 2023 and are projected to exceed 49 million units by 2029. Furthermore, Mordor Intelligence [2] estimates that the global electric bicycle market will grow from USD 34.98 billion in 2024 to USD 51.78 billion by 2029. Currently, the ownership of electric bicycles in China has exceeded 425 million units [3]. However, the rapid expansion of electric bicycle usage has been accompanied by an increasing number of fire incidents. According to China Daily/Asia News Network [4], approximately 18,000 and 21,000 electric bicycle fire accidents were reported in China in 2022 and 2023, respectively, posing a serious threat to public safety and property. Studies have shown that arc faults are a major contributor to these incidents, primarily resulting from loose electrical connections, insulation aging, or conductor damage. Arc discharge can generate extremely high temperatures within a very short period, which may ignite surrounding combustible materials and consequently lead to fire accidents. Therefore, the development of an effective arc fault identification method is of great importance for improving the operational safety of electric bicycles.

To improve electrical safety, extensive research has been conducted on arc fault detection and identification. Electrical characteristic analysis is one of the most widely used approaches. Chae et al. [5] proposed a fast Fourier transform (FFT)-based method for DC arc fault detection, while Benjamin et al. [6] introduced an online identification method based on the Hurst exponent for vehicle electrical systems. Frequency-domain analysis has also been widely applied because arc faults often generate distinctive spectral characteristics. Chen et al. [7] analyzed the frequency spectrum of electric bicycle DC arc faults, Xiao et al. [8] investigated low-voltage DC arc characteristics, and Hwang et al. [9] developed a time–frequency analysis method for AC series arc fault detection.

Signal processing and intelligent identification methods have further improved arc fault feature extraction and classification. Representative studies include harmonic analysis [10], signal-processing-based arc detection methods [11], noise-reduction-based feature extraction [12], and periodic background subtraction methods [13]. In addition, optical, electromagnetic, and sensor-based methods have been investigated to capture physical phenomena associated with arc discharge [14,15,16]. With the development of artificial intelligence, long short-term memory and transformer (LSTM-Transformer) models [17], explainable learning frameworks [18], machine learning-based fault diagnosis approaches [19,20], and hybrid machine learning methods [21] have also been applied to arc fault diagnosis. Moreover, feature-fusion and ensemble learning frameworks have been proposed to improve recognition performance under complex operating conditions [22,23,24].

Although these studies provide important references for arc fault detection, electric bicycle arc fault identification still faces several specific challenges. First, electric bicycles operate under frequent variations in state of charge (SoC), load torque, and motor speed, which cause strong nonstationarity in the current signal. Second, controller switching, motor commutation, and load disturbances may introduce waveform distortion and transient fluctuations that partially resemble arc fault signatures. Third, existing studies often focus on either DC-side or AC-side faults individually, while simultaneous identification of normal conditions, DC-side series arc faults, and AC-side series arc faults under multi-condition operation remains insufficiently investigated. Therefore, a lightweight and interpretable identification method suitable for multi-condition electric bicycle operation is still needed.

To address these challenges, this paper proposes an electric bicycle series arc fault identification method based on improved principal component analysis (PCA) and support vector machine (SVM). A multi-condition experimental platform is established to collect current signals under different SoC, torque, and speed conditions. Five time-domain features and three frequency-domain features are extracted to characterize arc fault behavior from multiple perspectives. An improved PCA-based feature fusion strategy is then employed to reduce feature redundancy while preserving arc-sensitive information. Finally, a radial basis function (RBF)-SVM classifier is developed to identify normal conditions, DC-side series arc faults, and AC-side series arc faults.

The remainder of this paper is organized as follows. Section 2 introduces the experimental platform and dataset construction. Section 3 presents the time–frequency analysis and feature extraction process. Section 4 describes the improved PCA-based feature fusion strategy, SVM classification model, and performance evaluation results. Section 5 discusses deployment feasibility, practical considerations, and limitations. Finally, Section 6 concludes the paper.

## 2. Experimental Platform

### 2.1. Experimental Platform Components

**Figure 1:**
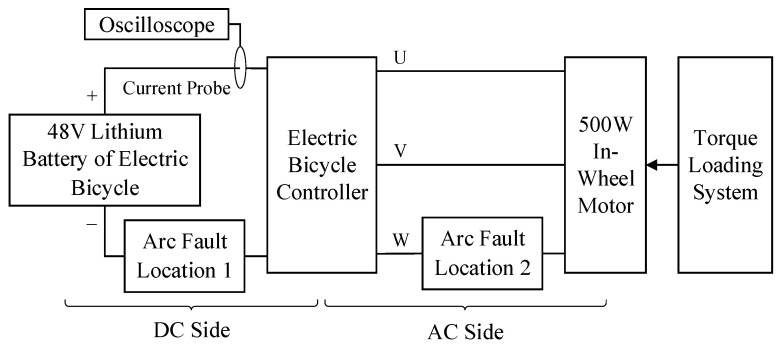
Architecture of the electric bicycle series arc fault experimental platform.

**Figure 2:**
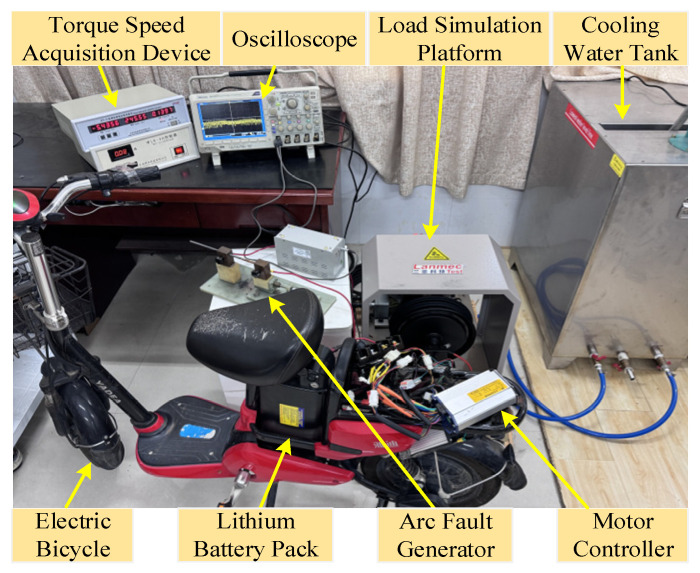
Physical setup of the electric bicycle series arc fault experimental platform.

The structural diagram of the electric bicycle series arc fault experimental platform is shown in Figure 1, and the physical setup is depicted in Figure 2.

In the platform, the DC-side arc fault is located between the battery pack and the controller input, while the AC-side arc fault is located between the controller output and the hub motor phase line. These positions correspond to practical connector and cable locations in electric bicycles where vibration, connector loosening, insulation aging, moisture, thermal cycling, mechanical stress, and charging/discharging cycles may initiate arc faults. The listed values in Table 1 are the operating levels used in the present experimental platform.

Unless otherwise specified, all current signals analyzed in this study were acquired from the DC-side measurement path located between the battery pack and the controller. For AC-side arc fault conditions, the fault was generated on the controller–motor path, while fault identification was still performed using the corresponding DC-side current response.

The platform can simulate normal and series arc fault states under various driving conditions. Its core components include a 48 V lithium-ion battery pack (Phylion Battery Co., Ltd., Suzhou, China), an electric bicycle controller, a 500 W hub motor, a laboratory-built series arc fault generator, signal acquisition equipment, and a torque loading system. The hub motor was integrated in the YADEA electric bicycle platform (Yadea Technology Group Co., Ltd., Wuxi, China). The electric bicycle controller was a commercial controller, model JK4860-A1-L, equipped with a matching S12G-12-L wiring harness supplied by Wuxi Saiying Power Technology Co., Ltd. (Wuxi, China). The signal acquisition equipment comprises a Keysight N2783B current probe (Keysight Technologies, Santa Rosa, CA, USA), a DPO4104B-L digital phosphor oscilloscope (Tektronix, Inc., Beaverton, OR, USA), and a CFY-10 torque–speed–power acquisition instrument (Jiangsu Lanmec Electromechanical Technology Co., Ltd., Hai’an, China). The torque loading system mainly consists of a CFC200 magnetic powder dynamometer (Jiangsu Lanmec Electromechanical Technology Co., Ltd., Hai’an, China), a WLK-3A stabilized-current power controller (Beijing Haibohua Technology Co., Ltd., Beijing, China), and a cooling water tank.

### 2.2. Experimental Scheme and Dataset

To systematically investigate the arc fault characteristics of electric bicycles under varying operating conditions, the experimental parameters outlined in Table 2 have been designed. By establishing four levels of SoC, seven levels of torque, and ten levels of speed, the experiment covers representative battery and load states for the 48 V electric bicycle platform.

Each current record was acquired for 400 ms at a sampling frequency of 25 kS/s, resulting in approximately 10,000 sampling points per record. The stratified training, validation, and test split preserves the class distribution across normal operation, DC-side arc faults, and AC-side arc faults, which improves the reliability of the held-out evaluation.

To ensure the scientific validity and effectiveness of algorithm training, validation, and testing, a stratified sampling strategy was used to divide the current dataset. The specific ratio is 7:1.5:1.5, meaning that the training set contains 1764 samples for model parameter optimization. The validation set contains 378 samples for tuning and early stopping of hyperparameters. The test set contains 378 samples for evaluating the final performance of the algorithm. The complete dataset contains 2520 samples, including normal-operation samples, DC-side series arc fault samples, and AC-side series arc fault samples collected under all operating-condition combinations.

## 3. Time–Frequency Analysis

### 3.1. Characteristic Comparison

Because the characteristics of series arc faults vary with operating conditions, the current signals of electric bicycles under different working conditions are analyzed in the time–frequency domain.

#### 3.1.1. Series Arc Fault State

**Figure 3:**
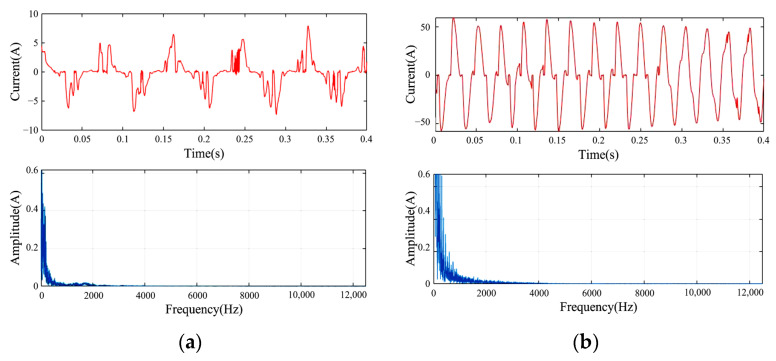

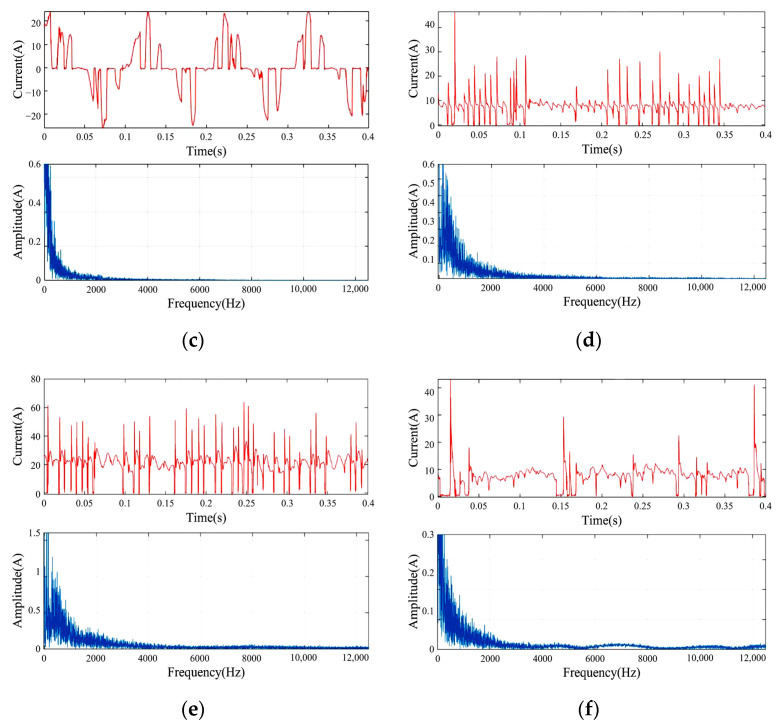
Time–frequency characteristics of representative arc fault conditions: (**a**) AC-side arc fault (low SoC, light torque, low speed); (**b**) AC-side arc fault (low SoC, heavy torque, low speed); (**c**) AC-side arc fault (high SoC, medium torque, low speed); (**d**) DC-side arc fault (low SoC, medium torque, low speed); (**e**) DC-side arc fault (low SoC, medium torque, high speed); (**f**) DC-side arc fault (high SoC, light torque, high speed).

The time–frequency characteristics of series arc faults are affected by operating conditions such as SoC, torque, and speed, as illustrated in Figure 3.

Under AC-side arc fault conditions, the measured DC-side current exhibits periodic zero-rest intervals at low SoC and more pronounced waveform mutations at high SoC. In contrast, DC-side arc faults are more sensitive to speed variations and produce stronger high-frequency spectral enhancement.

The above observations indicate that AC-side arc faults exhibit more pronounced time-domain distortions, such as non-periodic waveform deformation, zero-rest intervals, and flat-shoulder behavior. In contrast, DC-side arc faults tend to produce more evident frequency-domain enhancement, characterized by increased spectral peaks whose intensity generally increases with speed.

#### 3.1.2. Normal State

**Figure 4:**
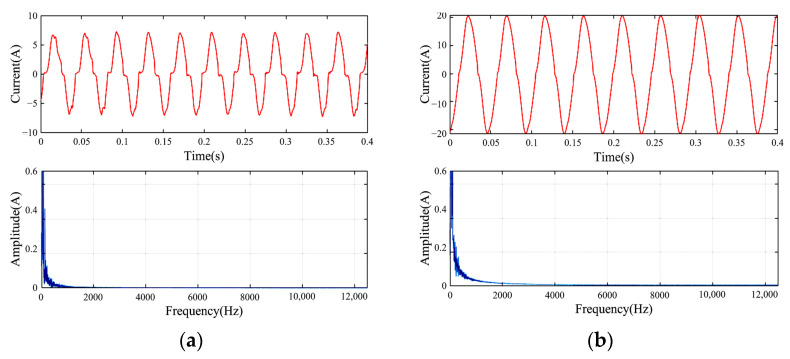
DC-side current characteristics under normal AC-side operating conditions: (**a**) low SoC; (**b**) high SoC.

**Figure 5:**
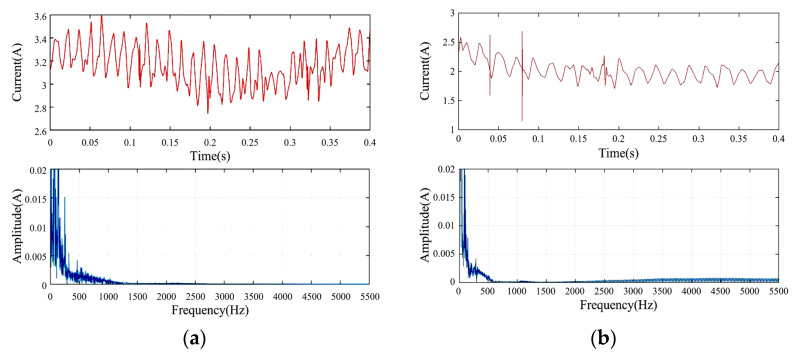
DC-side current characteristics under normal DC-side operating conditions: (**a**) low SoC; (**b**) high SoC.

The time–frequency characteristics under normal conditions are predominantly influenced by SoC, as shown in Figure 4 and Figure 5.

Under normal AC-side operating conditions, the waveform exhibits a flat-shoulder shape at low SoC, with frequency amplitude above 1 kHz approaching zero. At high SoC, the waveform approximates a standard sine wave. On the DC side, current amplitude is negatively correlated with SoC, with values of approximately 3.2 A at low SoC and 2.0 A at high SoC.

For practical riding conditions, normal acceleration and braking mainly appear as continuous low-frequency trend variations in current amplitude. In contrast, series arc faults are characterized by random spikes, intermittent breakdown, non-periodic distortion, high-frequency energy concentration, increased kurtosis, and increased spectral kurtosis. The 1.5–5 kHz FIR filter suppresses most low-frequency load variations while retaining high-frequency arc disturbances. Therefore, the subsequent multi-feature fusion and nonlinear SVM classification are used to reduce false alarms caused by normal transients.

The frequency-domain analysis shows that the characteristic frequency band of DC-side arc faults is mainly concentrated within 0–5 kHz, whereas that of AC-side arc faults is primarily distributed within 0–4 kHz. In contrast, the characteristic frequency band for the AC side of normal signals is predominantly in the range of 0–1.5 kHz, and for the DC side, it is mainly focused on 0–1 kHz. This indicates a partial overlap between the frequency domain characteristics of normal signals and arc faults, making it challenging to achieve accurate differentiation based solely on a single frequency band or time domain characteristics. Therefore, direct fault identification based solely on waveform observation or a single frequency-domain indicator may not be sufficiently reliable. To improve discrimination capability under complex operating conditions, multiple complementary time-domain and frequency-domain features are extracted in the following subsections.

### 3.2. Signal Preprocessing and Feature Extraction

Based on the above frequency-band differences, a finite impulse response filter is employed to process the original current signals, with the passband set to 1.5–5 kHz. This filtering range effectively extracts arc fault features while suppressing interference from most normal signals, thereby providing an optimized data foundation for subsequent multi-dimensional feature extraction.

#### 3.2.1. Time-Domain Feature Extraction

Multiple time-domain features are extracted based on the waveform distortion characteristics of arc faults. The root mean square (RMS) reflects effective power changes, the standard deviation (STD) reflects non-periodic fluctuations, the skewness (SK) reflects waveform asymmetry, the kurtosis (KUR) captures peak pulses, and the current amplitude (CA) reflects amplitude changes. The calculation formulas are as follows:

(1) $$\mathrm{RMS}=\sqrt{\frac{1}{N}\sum_{i=1}^{N}X_{i}^{2}}$$

(2) $$\mathrm{STD}=\sqrt{\frac{1}{N}{\sum_{i=1}^{N}\left(X_{i}-\mu \right)}^{2}}$$

(3) $$\mathrm{SK}=\frac{1}{N}{\sum_{i=1}^{N}\left(\frac{X_{i}-\mu }{\sigma }\right)}^{3}$$

(4) $$\mathrm{KUR}=\frac{1}{N}{\sum_{i=1}^{N}\left(\frac{X_{i}-\mu }{\sigma }\right)}^{4}-3$$

(5) $$\mathrm{CA}=\mathrm{MAX}\;\left(\left|X_{i}\right|\right)$$

where *μ* represents the mean, *σ* represents the standard deviation, *N* represents the total number of current samples, and *X_i_* represents the value of each data point in the current sample.

**Figure 6:**
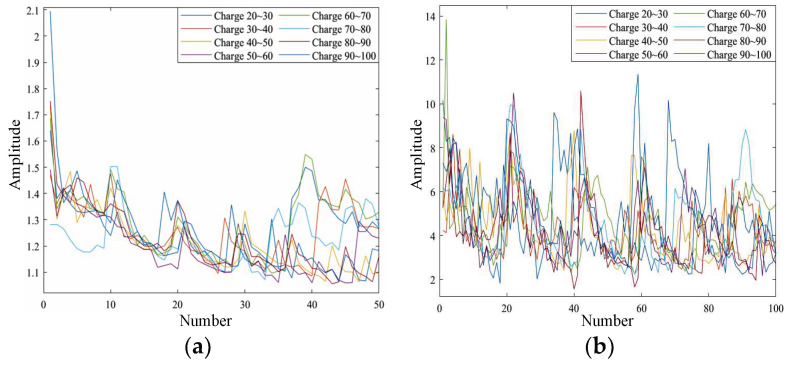

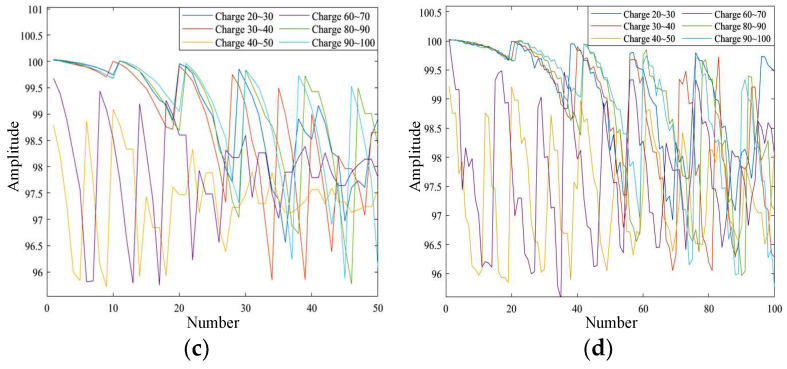
Comparison of current–amplitude features under different operating conditions: (**a**) normal DC-side operation; (**b**) DC-side arc fault; (**c**) normal AC-side operation; (**d**) AC-side arc fault.

**Figure 7:**
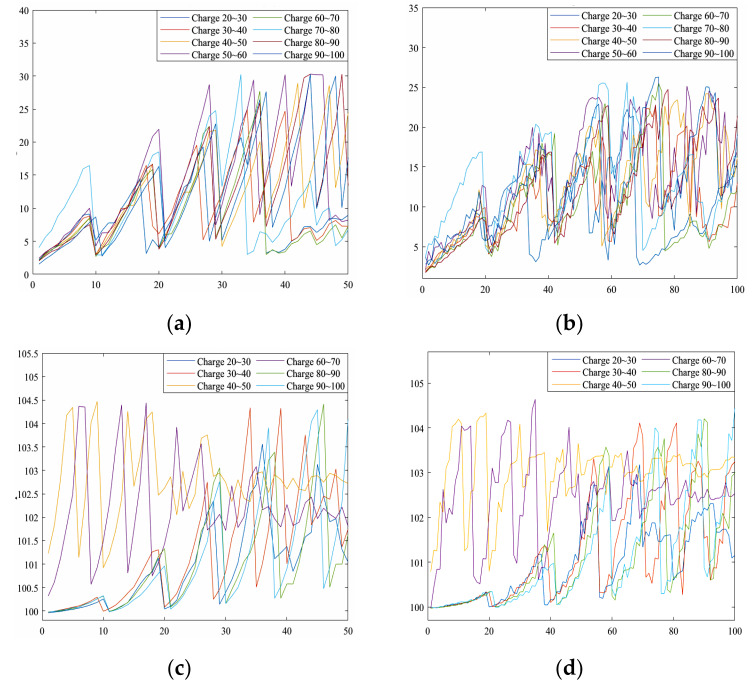
Comparison of RMS features under different operating conditions: (**a**) normal DC-side operation; (**b**) DC-side arc fault; (**c**) normal AC-side operation; (**d**) AC-side arc fault.

CA and RMS are analyzed in detail, with results shown in Figure 6 and Figure 7.

Although current amplitude reflects the overall magnitude variation caused by arc faults, it cannot fully characterize the irregular fluctuation behavior introduced by intermittent discharge. Therefore, RMS is further analyzed as a complementary descriptor of waveform energy variation.

The above observations demonstrate that time-domain features can effectively characterize amplitude fluctuation and waveform distortion. However, some normal and fault conditions still exhibit partial overlap. Therefore, frequency-domain features are further introduced to characterize spectral redistribution caused by arc discharge.

#### 3.2.2. Frequency-Domain Feature Extraction

Based on the spectral distortion characteristics of arc faults, multiple frequency-domain features are extracted. The amplitude–frequency energy (AFE) identifies high-frequency energy accumulation, the amplitude–frequency mean (AFM) reveals the shift of the spectral center of gravity, and the amplitude–frequency kurtosis (AFK) quantifies spectral steepness. The calculation formulas are as follows:

(6) $$\mathrm{AFE}={\sum_{i=1}^{N}\left|X(f_{i})\right|}^{2}$$

(7) $$\mathrm{AFM}=\frac{\sum_{i=1}^{N}f_{i}\cdot \left|X(f_{i})\right|}{\sum_{i=1}^{N}\left|X(f_{i})\right|}$$

(8) $$\mathrm{AFK}=\frac{\sum_{i=1}^{N}P{\left(f_{i}\right)}^{4}}{{\left(\sum_{i=1}^{N}P(f_{i})\right)}^{2}}-3$$

where *f_i_* is the frequency of the *i*-th frequency point, *X*(*f_i_*) is the Fourier transform amplitude at this frequency point, *P*(*f_i_*) is the power spectral density value at this frequency point, and *N* is the total number of frequency points.

**Figure 8:**
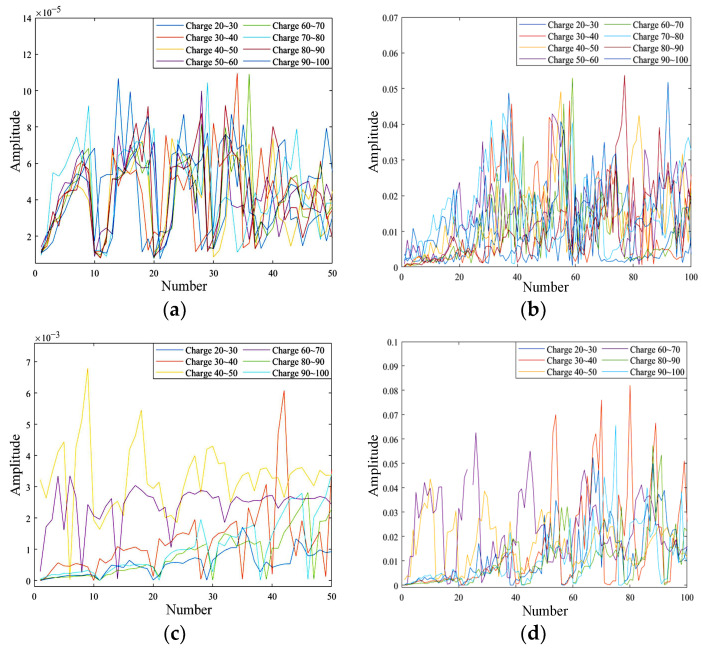
Comparison of amplitude–frequency mean features under different operating conditions: (**a**) normal DC-side operation; (**b**) DC-side arc fault; (**c**) normal AC-side operation; (**d**) AC-side arc fault.

**Figure 9:**
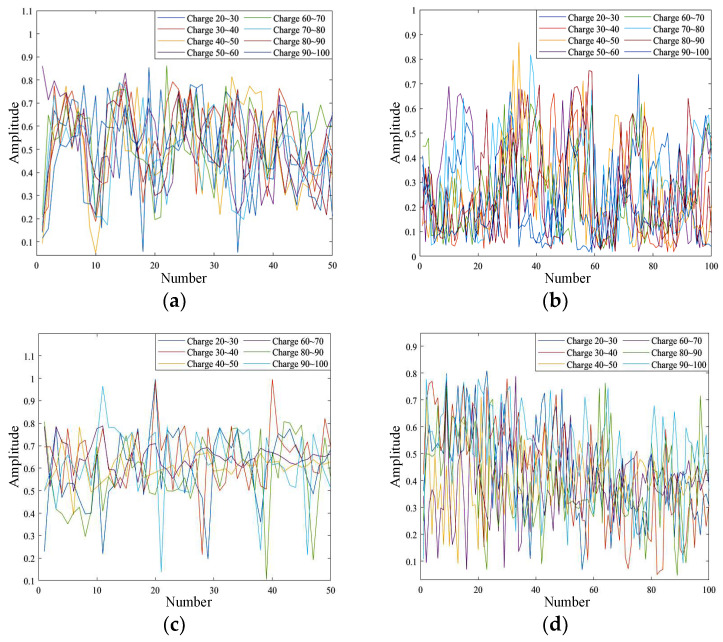
Comparison of amplitude–frequency kurtosis features under different operating conditions: (**a**) normal DC-side operation; (**b**) DC-side arc fault; (**c**) normal AC-side operation; (**d**) AC-side arc fault.

AFM and AFK are analyzed in detail, with results shown in Figure 8 and Figure 9.

Although the amplitude–frequency mean reflects the migration of spectral energy, it cannot fully describe the concentration degree of spectral peaks. Therefore, amplitude–frequency kurtosis is further analyzed.

This phenomenon indicates that series arc faults increase the asymmetry and concentration of the spectral energy distribution. The concentration of amplitude–frequency energy leads to an increase in both the amplitude–frequency mean and amplitude–frequency kurtosis.

The above results indicate that no single feature can reliably distinguish all operating conditions because partial overlap exists between normal and arc fault states. Therefore, the eight extracted time–frequency features are jointly used for feature fusion and classification. Their physical interpretation is summarized in Table 3.

DC-side arcs are more associated with high-frequency energy accumulation and spectral steepness, whereas AC-side arcs are more associated with waveform distortion, flat-shoulder behavior, and periodicity disruption. The combined time-domain and frequency-domain features therefore cover both fault mechanisms.

## 4. Improved PCA and SVM Identification Method

### 4.1. Feature Fusion via Improved PCA

To address the high dimensionality and redundancy of time–frequency domain features in series arc fault identification for electric bicycles, this paper proposes an improved PCA method that achieves feature fusion and dimensionality reduction by reconstructing the projection matrix. This method first standardizes the eight current features, analyzes the correlation between the original features and principal components, and then constructs a compact fused feature representation that preserves arc-sensitive principal-component information. The specific implementation process is as follows:

First, the feature matrix ***X*** ∈ **R**^n×8^ (where n is the sample count, and 8 is the original feature dimension) is standardized, and the covariance matrix cov(***X***) ∈ **R**^8×8^ is calculated:

(9) $$\mathrm{cov}(X)=\frac{1}{n-1}X^{T}X$$

Next, eigenvalue decomposition is performed on cov(***X***):

(10) $$\mathrm{cov}(X)=W\Lambda W^{T}$$

Since the covariance matrix is real symmetric, the eigenvector matrix ***W*** ∈ **R**^8×8^ satisfies ***W*** ^T^ ***W*** = ***I***, where ***W*** = [***w***_1_, ***w***_2_, …, ***w***_8_] and ***Λ*** ∈ **R**^8×8^ is the eigenvalue diagonal matrix.

**Figure 10:**
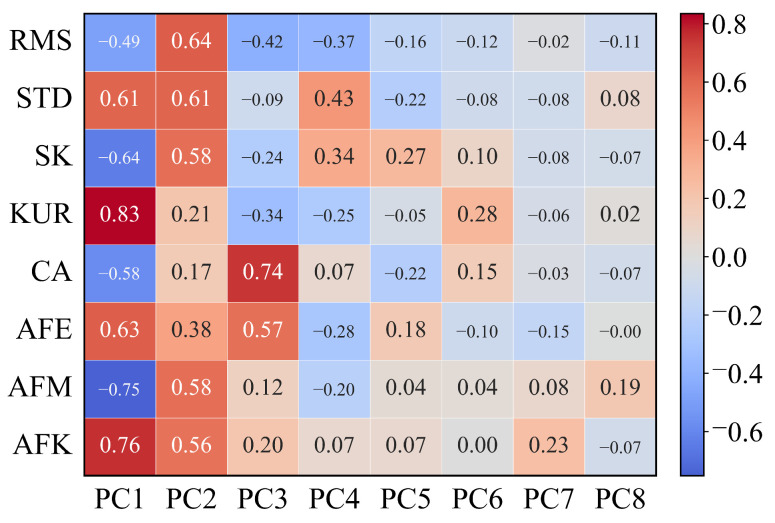
Correlation weights between original features and principal components.

In traditional PCA, dimensionality reduction is usually performed according to variance contribution. However, arc-sensitive information may not be concentrated exclusively in the dominant principal components. Therefore, the correlation distribution between the original features and the principal components was analyzed before feature fusion. As shown in Figure 10, the original time-domain and frequency-domain features exhibit different correlation strengths with the principal components.

Specifically, KUR, AFK, and AFE show strong positive correlations with PC1, indicating that PC1 mainly preserves impulsive discharge information and high-frequency energy concentration caused by arc ignition and extinction. In contrast, RMS, STD, SK, and AFM contribute significantly to PC2 and PC3, suggesting that these components capture waveform fluctuation, asymmetry, and spectral-center migration.

**Figure 11:**
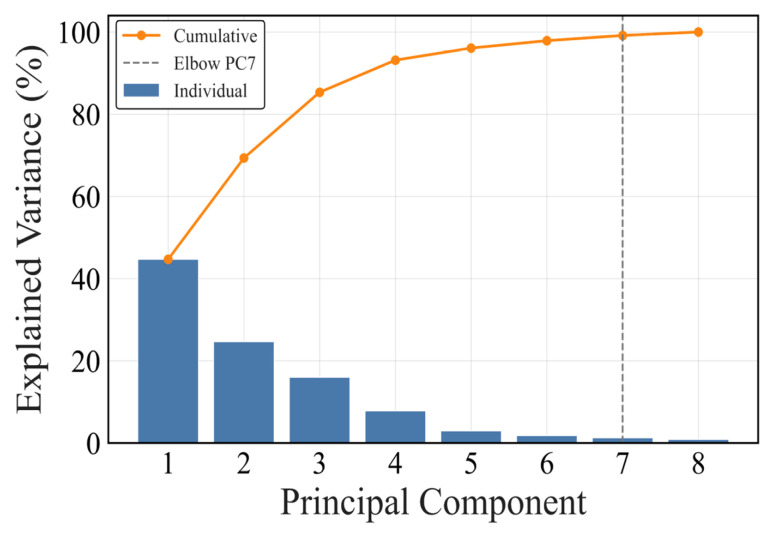
Explained variance ratio and cumulative contribution of principal components.

Figure 11 shows the explained variance ratio of each principal component together with the cumulative contribution curve.

The first three principal components account for approximately 85% of the total variance, while the cumulative contribution reaches approximately 98.8% at PC7. Although traditional PCA would typically retain only the first several principal components according to variance contribution, the correlation analysis shown in Figure 10 reveals that arc-sensitive spectral information is partially distributed among middle-order components. Therefore, variance contribution, feature correlation, and validation performance were jointly considered when designing the final PCA-fused representation.

To determine an appropriate fused representation, several PCA fusion strategies were evaluated and compared. The compared schemes differ in the manner in which middle-order principal components are retained or fused. Their classification performance, dimensionality, and computational efficiency are summarized in Table 4.

Among all compared strategies, the representation consisting of PC1, PC2, PC3, fused PC4–PC7, and PC8 achieved the best overall balance between classification performance and feature compactness. Therefore, this representation was selected as the final PCA-fused feature space.

After completing the feature decomposition, a new five-dimensional projection matrix ***W****_new_* ∈ **R**^8×5^ is constructed:

(11) $$W_{new}=[w_{1},w_{2},\frac{\sum_{i=3}^{6}\lambda_{i}w_{i}}{\sum_{i=3}^{6}\lambda_{i}},w_{7},w_{8}]$$

Here, *λ_i_* denotes the eigenvalue of the *i*-th principal component.

The fusion weights are determined according to the normalized eigenvalue contributions of the corresponding principal components, allowing components with higher variance contributions to retain greater influence in the fused representation.

Dimensionality reduction features are directly computed:

(12) $$Y_{new}=XW_{new}=\left[\mathrm{PC}1,\;\mathrm{PC}2,\;\mathrm{PC}3,\;\mathrm{fused}\;\mathrm{PC}4–\mathrm{PC}7,\;\mathrm{PC}5\right]$$

The columns of ***Y****_new_* ∈ **R**^n×5^ correspond to the final five fused principal-component features.

A comparison with traditional PCA is presented in Table 5.

As shown in Table 5, the proposed PCA-fused representation achieves higher classification performance than traditional PCA while maintaining a compact feature dimension. Compared with the traditional PCA scheme that retains only the first three principal components, the proposed method improves the test accuracy from 98.15% to 98.68%, increases the Macro-F1 score from 0.9815 to 0.9869, and improves the Macro-AUC from 0.9894 to 0.9931. These results indicate that the proposed fusion strategy effectively preserves arc-sensitive information contained in middle-order principal components while avoiding excessive feature dimensionality. Therefore, the resulting five-dimensional PCA-fused feature representation was selected as the input feature space for subsequent fault classification.

### 4.2. SVM Classification with Optimized Features

The optimized feature vector is subsequently used as the input of the SVM classifier. The core task is to train the weight vector ***ω*** to construct the decision function:

(13) $$f(X)=\omega^{T}\phi (X)+b$$

where ***X*** represents the input feature vector, ***ω*** is the weight vector (reflecting the weighting coefficient of each principal component after PCA dimensionality reduction in fault identification), *b* is the bias term (used to adjust the classification hyperplane), and *ϕ*(***X***) is the kernel function mapping, which maps the input feature space to a high-dimensional feature space to handle nonlinear separable problems.

To optimize the SVM’s classification performance, it is necessary to maximize the classification margin by solving for the optimal weight vector ***ω*** and bias term *b*. For the three classification problems of normal state, DC series arc fault, and AC series arc fault, an optimization model is established using the One-vs-Rest strategy. For the *k*-th class (*k* ∈ {0, 1, 2}), the optimization problem is solved:

(14) $$\begin{array}{c} \underset{\varpi ,b,\xi_{i}}{\min\;}\;\frac{1}{2}{\left\|\omega \right\|}^{2}+C\sum_{i=1}^{N}\xi_{i}\\ s.t.\;y_{i}\;(\omega^{T}\phi (\;x_{i})+b)\geq 1-\xi_{i},\;\xi_{i}\geq 0,\;i=1,2,\ldots ,N \end{array}$$

Here, *y_i_* ∈ {−1, +1} is the binary label of the sample. When the sample belongs to class *k*, it is labeled as +1; otherwise, it is labeled as −1. *C* is the penalty parameter, balancing the classification margin and misclassification loss. *ξ_i_* is a slack variable, allowing some samples to be within the classification margin, thereby enhancing the model’s fault tolerance to noise and abnormal samples.

Given the nonlinear characteristics of the series arc fault current features of electric bicycles, the RBF kernel is selected to complete the nonlinear mapping:

(15) $$K(x_{i},x_{j})=\exp(-\gamma {\left\|x_{i}-x_{j}\right\|}^{2})$$

Grid search parameters, *C* ∈ {0.1, 1, 10, 100}, *γ* ∈ {0.001, 0.01, 0.1, 1}, were employed with 5-fold cross-validation. This covers four orders of magnitude to balance efficiency and nonlinearity handling in RBF kernels. The optimal parameter combination was identified through grid search and validation, ensuring the model’s generalization performance.

The RBF-SVM classifier was selected according to the characteristics of the input feature representation. In this study, the classifier input is a low-dimensional PCA-fused feature vector derived from manually extracted time–frequency statistical features, rather than raw waveform sequences or images. Therefore, deep models such as convolutional neural networks (CNNs) and long short-term memory (LSTM), which are more suitable for spatial patterns or long temporal sequences, may introduce unnecessary model complexity and increase the risk of overfitting under the present feature-input condition.

Compared with deep learning models, RBF-SVM is well suited for medium-scale, low-dimensional, and nonlinear classification problems. It can construct nonlinear decision boundaries in the fused feature space while maintaining relatively low computational cost and good interpretability.

### 4.3. Performance Evaluation Results

To verify whether the selected PCA-SVM framework provides a reasonable tradeoff between recognition performance and computational complexity, several representative machine learning classifiers were further evaluated using the same data split and feature representation. The comparison results are summarized in Table 6.

**Figure 12:**
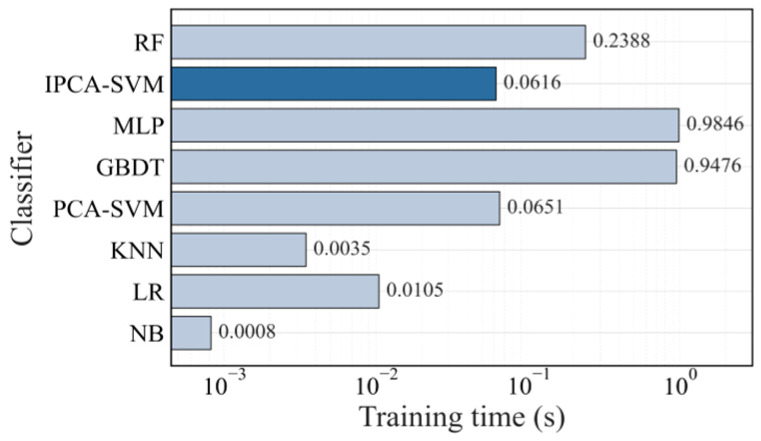
Training time comparison of different classifiers.

As shown in Table 6, Random Forest achieves the highest accuracy of 98.94%, while the proposed improved PCA-SVM achieves 98.68%, which is comparable to MLP and higher than GBDT, KNN, Logistic Regression, and Naive Bayes. Although Random Forest shows a marginal accuracy advantage, the proposed improved PCA-SVM maintains a simpler model structure and lower computational complexity. The training time comparison in Figure 12 further illustrates the computational advantage of the proposed framework.

MLP and Gradient Boosting require approximately 0.9 s of training time, whereas the proposed improved PCA-SVM requires only 0.0616 s while maintaining comparable recognition performance. Although KNN and Logistic Regression require shorter training time, their recognition accuracy is lower than that of the proposed method. Therefore, the improved PCA-SVM provides a balanced solution in terms of accuracy, training efficiency, and model interpretability.

**Figure 13:**
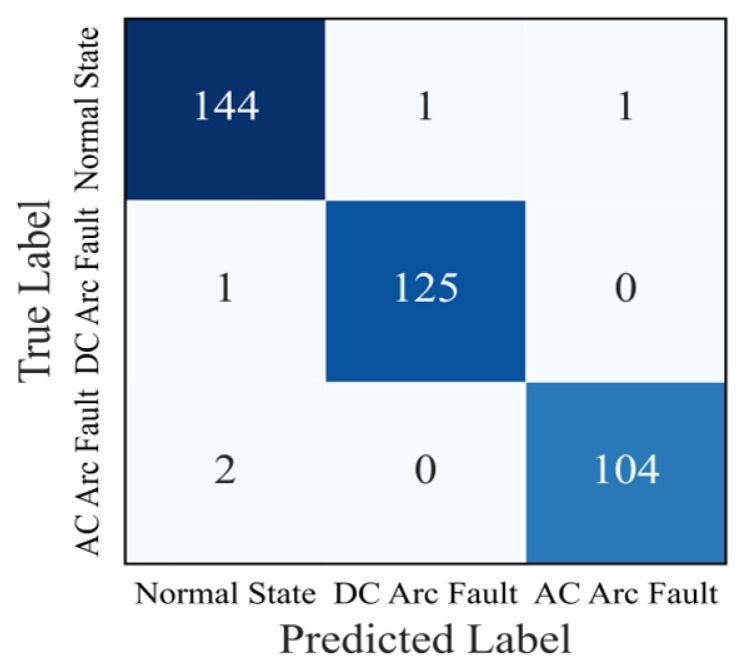
Confusion matrix of the proposed improved PCA-SVM model. The color intensity represents the number of samples in each cell.

To visually quantify the recognition algorithm’s performance on the test set, the confusion matrix is provided in Figure 13.

The results indicate that the correctly classified samples for the normal state, DC series arc fault, and AC series arc fault are 144, 125, and 104, respectively. Only five samples are misclassified among 378 test samples, corresponding to an overall test accuracy of 98.68%. The highest misclassification occurs between normal and AC-side arc fault samples, which is consistent with the waveform similarity observed in the time–frequency analysis. In contrast, mutual confusion between DC-side and AC-side arc faults remains limited, indicating that the extracted features preserve fault-type-specific information.

Based on the confusion matrix, core indicators were calculated:

(16) $$I_{a}=\frac{\sum TE_{i}}{\sum TE_{i}+\sum FN_{i}+\sum FP_{i}}$$

For *i* = 0, 1, 2, the true example (*TE_i_*) represents the number of samples correctly identified as category *i*; the false positive (*FP_i_*) represents the number of samples incorrectly identified as category *i*; and the false negative (*FN_i_*) represents the number of samples of category *i* incorrectly identified as another category.

**Figure 14:**
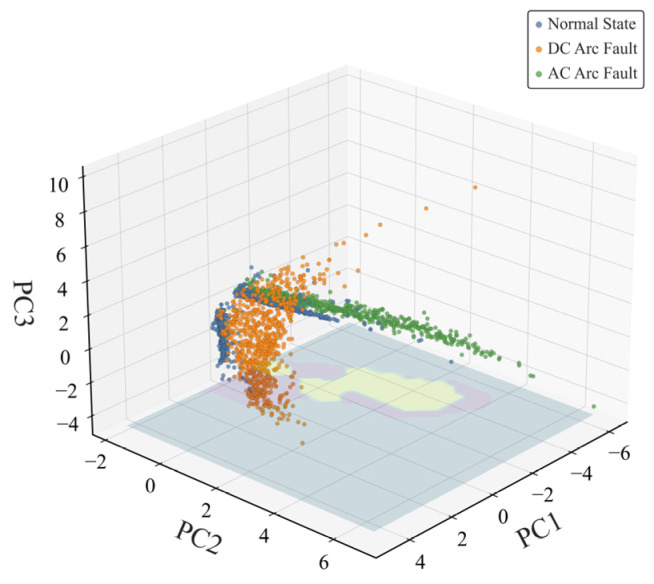
Three-dimensional visualization of the proposed PCA-fused feature space. Different colors denote different classes, and the translucent shadows indicate the projected distributions of the corresponding classes.

Figure 14 shows the distribution of the PCA-fused feature space. Although a small overlap remains near the class boundaries, the overall cluster separation demonstrates that the proposed representation effectively preserves class-discriminative information.

**Figure 15:**
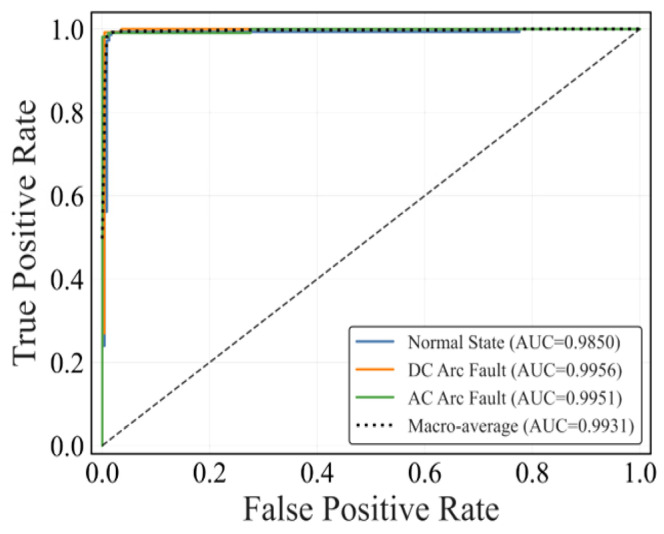
Multi-class ROC curves and corresponding AUC values.

The receiver operating characteristic (ROC) curve for the three categories was used to evaluate classification confidence, and the area under the curve (AUC) for each category was calculated, as shown in Figure 15.

The ROC analysis gives AUC values of 0.9850 for the normal state, 0.9956 for DC arc faults, and 0.9951 for AC arc faults, with a Macro-AUC of 0.9931. These results are consistent with the confusion matrix and indicate that the proposed feature-fusion and SVM classification strategy maintains high discrimination ability across different decision thresholds.

### 4.4. Deployment Feasibility and Model Interpretability

To further evaluate the practical applicability and interpretability of the proposed method, supplementary analyses were conducted from two perspectives: feature-level computational latency and feature importance. The latency analysis clarifies the software-side computational cost of the proposed framework, while the feature-importance analysis explains the relative contribution of the extracted time-domain and frequency-domain descriptors.

#### 4.4.1. Feature-Level Latency Analysis

Table 7 reports the feature-level computational latency of the proposed framework, including standardization, PCA fusion, and SVM inference. Hardware acquisition and communication delays are not included.

All signal processing, feature extraction, PCA fusion, SVM training, and performance evaluation were implemented in Python 3.12.7 using PyCharm 2024.3.4 (JetBrains, Prague, Czech Republic), together with NumPy 1.26.4, SciPy 1.13.1, scikit-learn 1.5.1, and Matplotlib 3.9.2.

#### 4.4.2. Feature Importance Analysis

**Figure 16:**
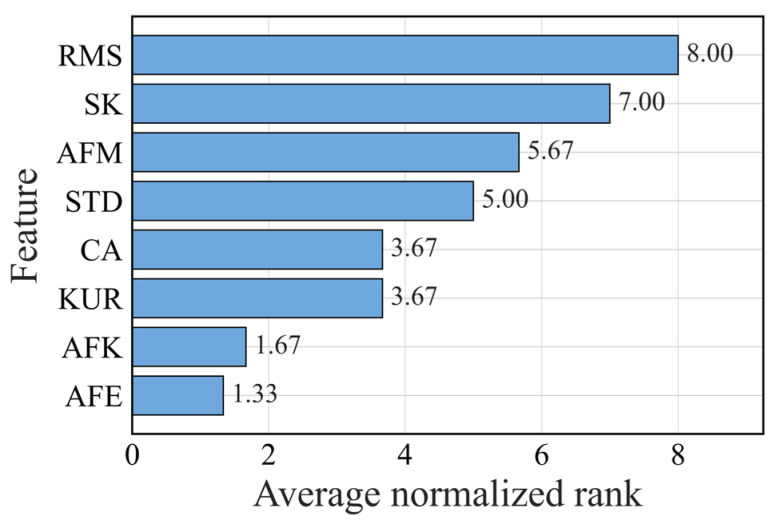
Relative importance ranking of the extracted time–frequency features.

In addition to computational efficiency, feature interpretability is also important for understanding why the proposed framework can distinguish normal conditions, DC-side arc faults, and AC-side arc faults. Therefore, the relative importance of the extracted features was further analyzed. Figure 16 presents the average normalized importance ranking of the extracted features.

RMS and SK obtain the highest rankings, indicating that waveform-energy variation and asymmetry provide the strongest discriminative information for arc fault identification. AFM and STD also contribute significantly, reflecting the importance of spectral-center migration and fluctuation intensity.

In contrast, AFE and AFK exhibit relatively lower rankings. However, these features still provide complementary information regarding high-frequency energy accumulation and spectral sharpness. The ranking results therefore support the use of multi-feature fusion rather than relying on any individual feature.

Future work should further investigate the influence of battery aging, environmental disturbances, and cross-platform operating conditions on feature importance and model generalization.

## 5. Practical Discussion

### 5.1. Deployment Scenario

The proposed method is intended primarily for embedded electric bicycle safety-monitoring applications after hardware implementation, rather than solely for offline diagnosis. In the present study, fault identification is performed using current signals acquired from the battery–controller DC path. Therefore, only a DC-side current sensor is required for implementation. After signal acquisition and preprocessing, the extracted feature vector can be projected into the optimized PCA-fused feature space and subsequently classified by the RBF-SVM model.

Because the proposed framework relies on low-dimensional statistical features and an RBF-SVM classifier, its computational requirements remain relatively low. Furthermore, only a single DC-side current sensor is required, which further supports deployment on embedded electric bicycle controllers.

### 5.2. Difference Between Normal Riding Transients and Arc Faults

Normal acceleration and braking mainly appear as continuous load-induced variations in the current envelope, and their energy is usually concentrated in relatively low-frequency components. In contrast, series arc faults originate from intermittent breakdown and unstable contact, producing random spikes, non-periodic distortion, zero-rest or flat-shoulder phenomena, and high-frequency energy enhancement. Therefore, normal riding transients and arc faults may partially resemble each other in the time domain, but they differ in impulsiveness, spectral distribution, and waveform irregularity.

### 5.3. Limitations and Future Work

Although the proposed method achieves high recognition accuracy under the tested multi-condition platform, several limitations remain. First, the experimental platform is based on a 48 V, 500 W class laboratory electric bicycle system. Different voltage platforms, motor powers, controller types, wiring harness structures, and sampling frequencies may change the current–signal distribution. Second, the current evaluation is based on offline extracted features, and complete end-to-end embedded latency, including acquisition, filtering, online feature extraction, communication, and protection-device actuation, has not yet been fully evaluated.

Battery state of health (SoH) was not independently controlled for in the present study. Since battery aging may increase internal resistance, voltage sag, thermal stress, and contact instability, different SoH levels may influence both arc fault occurrence probability and the statistical distribution of extracted features. Therefore, future work will focus on real-road experiments, embedded implementation, cross-platform validation, SoH-controlled tests, and generalization evaluation under vibration, temperature, humidity, electromagnetic interference, and long-term operating conditions.

## 6. Conclusions

This paper proposed an electric bicycle series arc fault identification method based on improved PCA and SVM. A multi-condition experimental platform covering different SoC, torque, and speed levels was established to collect current signals under normal operation, DC-side arc faults, and AC-side arc faults. Five time-domain features and three frequency-domain features were extracted to characterize arc fault behavior from multiple perspectives.

To reduce feature redundancy while preserving arc-sensitive information, an improved PCA-based feature fusion strategy was developed. The final feature representation consists of PC1, PC2, PC3, fused PC4–PC7, and PC8. Based on the optimized feature space, an RBF-SVM classifier was trained to distinguish normal conditions, DC-side arc faults, and AC-side arc faults. Experimental results demonstrate that the proposed method achieves 98.68% test accuracy, 0.9869 Macro-F1, and 0.9931 Macro-AUC. Compared with traditional PCA-SVM, the proposed framework improves classification accuracy by 0.53 percentage points while maintaining a compact feature representation and a feature-level latency of only 0.0180 ms/sample.

The additional classifier comparison, latency analysis, and deployment discussion demonstrate that the proposed improved PCA-SVM framework provides a practical balance among recognition accuracy, computational efficiency, interpretability, and deployment feasibility. Therefore, it represents a promising lightweight solution for electric bicycle arc fault monitoring and protection applications. Future work will focus on embedded implementation, cross-platform validation, battery aging analysis, and real-road verification under practical operating conditions.

## Figures and Tables

**Table 1:** Specifications of the experimental platform components.

Component	Model/Specification	Operating Parameter	Function in Platform
Lithium-ion battery pack	48 V lithium-ion battery pack (Phylion Battery Co., Ltd., Suzhou, China)	Used SoC levels: 20–40%, 41–60%, 61–80%, and 81–100%	Power supply
Hub motor	500 W class hub motor integrated in the YADEA electric bicycle platform (Yadea Technology Group Co., Ltd., Wuxi, China)	Used as the electric bicycle drive load	Drive load for current-signal generation
Electric bicycle controller	Commercial electric bicycle controller, model JK4860-A1-L, with matching S12G-12-L wiring harness supplied by Wuxi Saiying Power Technology Co., Ltd. (Wuxi, China)	Motor control and power conversion	Motor control and power conversion
Series arc fault generator	Laboratory-built series arc generator (Huaqiao University, Xiamen, China)	Controlled series arc faults on DC-side and AC-side paths	Generation of repeatable series arc fault conditions
Current probe	Keysight N2783B current probe (Keysight Technologies, Santa Rosa, CA, USA)	Measurement object: current signal	Current acquisition
Oscilloscope	DPO4104B-L digital phosphor oscilloscope (Tektronix, Inc., Beaverton, OR, USA)	Sampling frequency: 25 kS/s	Signal recording
Torque–speed–power instrument	CFY-10 torque–speed–power acquisition instrument (Jiangsu Lanmec Electromechanical Technology Co., Ltd., Hai’an, China)	Torque, speed, and power monitoring	Operating-condition monitoring
Torque loading system	CFC200 magnetic powder dynamometer (Jiangsu Lanmec Electromechanical Technology Co., Ltd., Hai’an, China) and WLK-3A stabilized-current power controller (Beijing Haibohua Technology Co., Ltd., Beijing, China)	Used torque levels: 5, 10, 15, 20, 25, 30, and 35 N·m	Load torque simulation
Speed settings	Experimental speed levels controlled by the torque loading system	50–500 r/min	Multi-speed operating-condition simulation

**Table 2:** Multi-condition experimental design.

Experimental Variable	Parameter Ranges
SoC (%)	20–40, 41–60, 61–80, 81–100
Torque (N·m)	5, 10, 15, 20, 25, 30, 35
Speed (r/min)	50, 100, 150, 200, 250, 300, 350, 400, 450, 500

**Table 3:** Physical interpretation of extracted time–frequency features.

Feature	Symbol	Domain	Physical Meaning	Arc-Related Characteristic
Root mean square	RMS	Time domain	Effective current and energy variation	Current/energy disturbance, load variation, and arc-related amplitude change
Standard deviation	STD	Time domain	Non-periodic fluctuation intensity	Unstable current fluctuation caused by intermittent arc discharge
Skewness	SK	Time domain	Waveform asymmetry	Asymmetric arc bursts and half-cycle distortion
Kurtosis	KUR	Time domain	Impulsive spikes and intermittent breakdown	Spike-like arc disturbance and abnormal peak concentration
Current amplitude	CA	Time domain	Instantaneous amplitude variation	Transient amplitude disturbance under arc ignition and extinction
Amplitude–frequency energy	AFE	Frequency domain	High-frequency energy accumulation	High-frequency energy enhancement introduced by series arc discharge
Amplitude–frequency mean	AFM	Frequency domain	Frequency-domain center shift	Spectral center migration caused by switching and arc disturbance
Amplitude–frequency kurtosis	AFK	Frequency domain	Sharpness of spectral distribution	Concentrated spectral peaks and arc-sensitive spectral steepness

**Table 4:** Performance comparison of different PCA fusion strategies.

Method	Feature Representation	Feature Count	Validation Accuracy (%)	Test Accuracy (%)	Macro-F1	Macro-AUC	Inference Time (ms/Sample)
Traditional PCA	PC1, PC2, PC3	3	96.30	98.15	0.9815	0.9894	0.0163
Fusion Scheme A	PC1, PC2, fused PC3–PC8	3	91.01	94.44	0.9436	0.9874	0.0327
Fusion Scheme B	PC1, PC2, fused PC3–PC5, fused PC6–PC8	4	96.03	97.62	0.9761	0.9926	0.0291
Fusion Scheme C	PC1, PC2, fused PC3–PC7, PC8	4	96.56	98.15	0.9815	0.9893	0.0206
Fusion Scheme D	PC1, PC2, PC3, fused PC4–PC8	4	97.09	98.41	0.9842	0.9914	0.0164
Fusion Scheme E	PC1, PC2, fused PC3–PC6, PC7, PC8	5	97.62	97.62	0.9759	0.9971	0.0173
Proposed PCA Fusion	PC1, PC2, PC3, fused PC4–PC7, PC8	5	98.15	98.68	0.9869	0.9931	0.0145
All PCs Separate	PC1–PC8	8	97.62	98.68	0.9869	0.9951	0.0159

**Table 5:** Performance comparison between traditional PCA and improved PCA.

Method	Feature Representation	Feature Count	Test Accuracy (%)	Macro-F1	Macro-AUC
Traditional PCA	PC1, PC2, PC3	3	98.15	0.9815	0.9894
Improved PCA	PC1, PC2, PC3, fused PC4–PC7, PC8	5	98.68	0.9869	0.9931
Difference	Improved minus traditional	2	+0.53	+0.0054	+0.0025

**Table 6:** Performance comparison of different classifiers.

Classifier	Accuracy (%)	F1-Score	Macro-AUC	Training Time (s)	Inference Time (ms/Sample)
Random Forest	98.94	0.9894	0.9949	0.2327	0.2109
Proposed Improved PCA-SVM	98.68	0.9869	0.9931	0.0612	0.0151
MLP	98.68	0.9868	0.9979	0.9014	0.0017
Gradient Boosting	98.41	0.9841	0.9959	0.9321	0.0064
Traditional PCA-SVM	98.15	0.9815	0.9894	0.0637	0.0164
KNN	97.88	0.9788	0.9932	0.0011	0.0236
Logistic Regression	97.62	0.9762	0.9924	0.0066	0.0005
Naive Bayes	83.60	0.8340	0.9821	0.0007	0.0010

**Table 7:** Feature-level computational latency analysis.

Module	Operation	Latency (ms/Sample)	Remark
Feature loading	Load extracted 8-D feature vector	0.0001	Extracted features are used as the input.
Standardization	Apply z-score standardization	0.0020	Z-score scaling before PCA and SVM.
PCA transform/fusion	Project and fuse selected PCs	0.0020	Principal-component projection and improved PCA fusion.
SVM inference	Predict class using RBF-SVM	0.0139	RBF-SVM prediction.
Total feature-level latency	Sum of feature-level operations	0.0180	Feature-level latency only; acquisition/filtering are excluded.

## Data Availability

The datasets generated and analyzed during the current study are available from the corresponding author upon reasonable request.

## Abbreviations

The following abbreviations are used in this manuscript:

PCA	principal component analysis
SVM	support vector machine
BMS	battery management system
RBF	radial basis function
AUC	area under the curve
SoC	state of charge
SoH	state of health
LSTM	long short-term memory
LSTM-Transformer	long short-term memory and transformer
CNN	convolutional neural network
FFT	fast Fourier transform
RMS	root mean square
STD	standard deviation
SK	skewness
KUR	kurtosis
CA	current amplitude
AFE	amplitude–frequency energy
AFM	amplitude–frequency mean
AFK	amplitude–frequency kurtosis
ROC	receiver operating characteristic
